# Comorbidity Burden in Lung Cancer and Malignant Pleural Mesothelioma: Nationwide Database Results of Turkey

**DOI:** 10.3390/medicina62050845

**Published:** 2026-04-29

**Authors:** Çiğdem Özdilekcan, Tarkan Özdemir, Mustafa Hamidullah Türkkanı, Naim Ata, Mesut Akyol, Mevlüt Karataş, Aslıhan Gürün Kaya, Aydın Yılmaz, Akın Kaya, Şuayip Birinci

**Affiliations:** 1 Department of Pulmonology, University of Health Sciences, Dr. Abdurrahman Yurtaslan Ankara Oncology Education and Research Hospital, 06200 Ankara, Türkiye; 2 Department of Pulmonology, Faculty of Medicine, Ankara Yıldırım Beyazıt University, Ankara City Hospital, 06800 Ankara, Türkiye; 3 Department of Strategy, Ministry of Health, 06100 Ankara, Türkiye; 4 Department of Biostatistics, Faculty of Medicine, Ankara Yıldırım Beyazıt University, 06800 Ankara, Türkiye; 5 Department of Pulmonology, Ankara Occupational and Environmental Diseases Hospital, 06280 Ankara, Türkiye; 6 Department of Pulmonology, Faculty of Medicine, Ankara University, 06800 Ankara, Türkiye; 7 Department of Pulmonology, University of Health Sciences, Ankara Atatürk Sanatorium Education and Research Hospital, 06010 Ankara, Türkiye; 8 Republic of Turkey, Ministry of Health, 06100 Ankara, Türkiye

**Keywords:** lung cancer, malignant pleural mesothelioma, comorbidity

## Abstract

*Background and Objectives:* The presence of comorbidities in both the pre- and post-diagnostic periods is a critical consideration in the diagnosis and management of patients with cancer. This study aimed to investigate the prevalence and burden of pulmonary and extrapulmonary comorbidities in patients diagnosed with lung cancer (LC) and malignant pleural mesothelioma (MPM). *Materials and Methods:* The data were obtained from official patient records of the Turkish Ministry of Health. Patients diagnosed with either lung cancer (LC) or malignant pleural mesothelioma (MPM) between 2015 and 2018 were included in the study. Comorbidities were classified as pulmonary or extrapulmonary. *Results:* A total of 74,835 patients with LC and 1678 patients with MPM were included. The burden of comorbid conditions increased significantly in the post-diagnostic period in both males and females across both cancer types. When the two cancer groups were compared with respect to diagnostic periods, comorbidities such as hypertension (HT), phlebitis/venous thrombosis/thrombophlebitis, pulmonary embolism, pneumothorax, and pleural effusion were significantly more prevalent in the MPM group (*p* < 0.05). Compared with the pre-diagnostic period, the comorbidity risk in LC was highest for pulmonary embolism, ARF, and pneumonia in the post-diagnostic period, whereas renal failure was the most frequent comorbidity in the MPM group (*p* < 0.001 and *p* = 0.024). When comparing changes in comorbidity burden between sexes in the lung cancer group, male patients had higher frequencies of pulmonary embolism, pneumonia, pneumothorax, and coronary artery disease than females. In contrast, in the female lung cancer group, the prevalence of chronic renal failure was higher than in males (OR = 2.14 vs. 2.00), whereas acute renal failure was more prominent in the male patient group (OR = 2.64 vs. 1.94). In gender-based comparison of comorbid conditions among patients with MPM, the risk of renal failure was higher in females than in males (CRF and ARF respectively: OR = 2.63 vs. 2.16 and OR = 6.80 vs. 5.44). Additionally, increased rates of COPD were observed in male patients within this group (OR = 1.93 vs. 1.81). *Conclusions:* Patients with LC and MPM are burdened not only by their primary malignancies but also by a wide spectrum of comorbidities, particularly in the post-diagnostic period. Comprehensive knowledge of comorbid conditions is essential for clinicians to guide clinical decision-making, anticipate disease progression, and optimize treatment strategies, thereby informing national healthcare policies. Future studies incorporating matched control groups or longitudinal designs with standardized surveillance protocols may help conduct better research.

## 1. Introduction

Recent data indicate that lung cancer (LC) remains one of the most common malignancies worldwide, associated with substantial morbidity and mortality both nationally and globally. It continues to represent a major public health challenge, imposing a significant socioeconomic burden. According to the statistical data of GLOBOCAN (Global Cancer Observatory), lung cancer was reported as the most frequently diagnosed cancer in 2022, responsible for almost 2.5 million new cases, or one in eight cancers worldwide (12.4% of all cancers globally) [1].

Cigarette smoking is the most well-established risk factor for the development of LC. Since the first publication of the U.S. Surgeon General’s report on smoking and health in 1964, the incidence of LC has declined significantly, in parallel with reductions in smoking prevalence [2].

According to the GLOBOCAN 2020 registry, lung cancer (LC) comprises 17.6% of all cancer types. It ranks highest in Turkey, with 41,000 new cases in 2020 and an age-adjusted incidence rate of 41.7 and 8.7 per 100,000 for men and women, respectively [3].

Malignant pleural mesothelioma is a neoplastic disease originating from the pleural mesothelium, with poor treatment options and poor survival. The main cause is exposure to mineral fibers, such as asbestos and erionite [4]. Environmental exposure to mineral fibers occurs in many countries, and erionite as a cause of mesothelioma has been well documented in studies of three villages in the Cappadocian region of Turkey, with an extraordinarily high mesothelioma incidence, particularly in the village of Karain. These villagers had the highest risk of mesothelioma in the world, with an annual incidence per 100,000 persons of 639 for men and 1267 for women [5].

In addition, several cofactors have been implicated in the pathogenesis of asbestos-related mesothelioma, including genetic predisposition, diets low in fruits and vegetables, viral factors, immune dysfunction, and recurrent serosal inflammation [6]. Previous studies have shown that major comorbid conditions are independent predictors of survival in patients undergoing radical surgery for MPM [7].

As originally defined by Feinstein, “comorbidity” refers to “any distinct additional clinical entity that has existed or may occur during the clinical course of a patient who has the index disease under study” [6,7]. In this context, comorbidity describes the coexistence of one or more chronic conditions alongside a primary disease of interest. The presence of comorbidities in patients with lung cancer may adversely affect prognosis. The symptoms of comorbid conditions may prompt patients to seek medical attention earlier, potentially leading to earlier diagnosis [8,9].

Conversely, in the presence of additional chronic diseases, symptoms of malignancy may be misattributed to pre-existing conditions, resulting in delayed diagnosis. Following diagnosis, comorbidities may also influence both the timing and outcomes of treatment. There is substantial evidence indicating that patients with comorbidities are less likely to receive curative treatments than those without [10].

Both LC and MPM impose a significant economic burden on patients, healthcare systems, and society due to their high prevalence and the costs associated with diagnosis, treatment, and palliative care. The presence of comorbidities may further complicate clinical decision-making and increase healthcare utilization. We hypothesized that patients with LC and MPM have a substantial burden of comorbid conditions present in the pre-diagnostic period, which further increases following diagnosis, and that the overall comorbidity burden differs between LC and MPM. Lung cancer (LC) and malignant pleural mesothelioma (MPM) were investigated together in this study due to their shared origin as thoracic malignancies, overlapping clinical presentations, and common exposure-related risk factors, particularly smoking and asbestos. Despite these similarities, they differ substantially in their pathogenesis, natural history, and treatment strategies. Comparing LC and MPM within the same population provides a unique opportunity to explore whether comorbidity burden reflects shared risk exposures or disease-specific characteristics, and to identify potential differences in comorbidity patterns that may have implications for clinical management. Despite growing evidence on the impact of comorbidities in thoracic malignancies, several important gaps remain. Most previous studies have focused on comorbidity burden at a single time point, without distinguishing between pre- and post-diagnostic periods. In addition, comparative data between LC and MPM are limited, particularly within the same population.

This nationwide study primarily aimed to investigate the prevalence of comorbid conditions in patients with LC and MPM using electronic medical records obtained from the Turkish Ministry of Health (MoH). Comorbidities were evaluated and compared across the pre- and post-diagnostic periods by time of onset. The secondary objective was to assess the overall burden of comorbid disease by comparing patients with LC and those with MPM.

## 2. Materials and Methods

### 2.1. Data Sources and ICD-10 Classification of Comorbidities

The data were derived from official patient records of the Turkish MoH. The descriptive data presented in this study were extracted by a multidisciplinary team consisting of a data miner, a statistician, and a physician, within a time frame determined by the MoH and in accordance with predefined research queries.

Patients diagnosed with either LC or MPM were included in this retrospective study. All included patients were diagnosed in 2016. The period from 1 January 2015, to the date of diagnosis in 2016 was defined as the pre-diagnostic period. The period from the date of diagnosis in 2016 to 31 December 2018, was defined as the post-diagnostic period.

Comorbidity data were analyzed for both pre- and post-diagnostic periods, including primary and secondary diagnoses recorded during both inpatient and outpatient admissions. The study population consisted of adults aged ≥18 years; pediatric patients were excluded. The dataset utilized in this study consists of aggregated summary counts (which are non-linkable summary statistics) provided by the Ministry of Health. Consequently, as individual-level raw data were not accessible, performing person-level matched analyses or multivariable adjustment for confounders like smoking status and clinical stage was not feasible.”

The ICD-10 codes used to identify comorbidities are listed below, according to the Coding algorithms for defining comorbidities in ICD-9-CM and ICD-10 administrative data [11]:

Lung cancer: C34 and subcategories
Mesothelioma: C45 and subcategories
Pulmonary embolism: I26 and subcategories
Chronic obstructive pulmonary disease (COPD): J44 and subcategories
Asthma: J45 and subcategories
Pneumothorax: J93 and subcategories
Pleural effusion: J90, J91
Pneumonia: J12 and subcategories; J13, J15 and subcategories; J16 and subcategories; J17 and subcategories; J18 and subcategories
Atherosclerotic heart disease: I25 and subcategories
Diabetes mellitus: E10 and subcategories; E11 and subcategories
Hypertension: I10 and subcategories
Chronic kidney failure: N18 and subcategories
Acute kidney failure: N17 and subcategories
Phlebitis, thrombophlebitis, and venous thrombosis: I80 and subcategories; I81, I82 and subcategories.

### 2.2. Data Validation and Cleaning

All diagnoses were retrieved from the Health Information Management System based on ICD-10 coding. Conditions recorded as pre-diagnoses or differential diagnoses were excluded from the analysis. Only diagnoses coded as confirmed were included. All diagnoses for LC and MPM were confirmed histopathologically, and only cases with definite diagnoses were included in the study. Comorbidity assessment was based on extrapulmonary and pulmonary diagnoses recorded in the Ministry of Health database, all of which had been confirmed by the patients’ primary or consulting physicians.

### 2.3. Definition of Comorbidities

The most prevalent general and disease-specific comorbidities were identified from the study population.

Comorbid conditions were defined as follows:

(i) Pulmonary comorbidities: Conditions potentially associated with LC and MPM, including pulmonary embolism (PE), deep venous thrombosis (DVT), pleural effusion, pneumothorax, pneumonia, chronic obstructive pulmonary disease (COPD), and asthma.
(ii) Extrapulmonary comorbidities: Conditions not directly related to the primary disease of interest, including diabetes mellitus (DM), hypertension (HT), acute renal failure (ARF), chronic renal failure (CRF), and coronary artery disease (CAD).

Classification According to Diagnostic Period:

Comorbidities were further categorized based on the timing of diagnosis:

(i) Pre-diagnostic comorbidity: Represents the baseline burden of comorbid disease prior to cancer diagnosis.
(ii) Post-diagnostic comorbidity: Represents the burden of comorbid disease following diagnosis, reflecting the disease course.

### 2.4. Statistical Analysis

Descriptive statistics were expressed as mean ± standard deviation (SD) for continuous variables and as number (percentage) for categorical variables. Chronic disease rates before and after cancer diagnosis were compared using the McNemar test for paired categorical data.

Odds ratios (ORs) and 95% confidence intervals (CIs) were calculated to reflect associations between cancer diagnosis and the risk of chronic diseases (crude/unadjusted comparisons without additional clinical covariates). The Chi-square test was used to compare the prevalence of chronic diseases between cancer types (ICD-10 C34 vs. C45).

All statistical analyses were performed using IBM SPSS Statistics for Windows, Version 22.0 (IBM Corp., Armonk, NY, USA). A *p*-value of <0.05 was considered statistically significant.

Sex-adjusted binomial regression models were implemented in Python (using Pandas, NumPy, Statsmodels, and Matplotlib libraries) to compare post-diagnostic comorbidities between MPM (C45) and LC (C34). The models were specified as generalized linear models (GLMs) with a binomial distribution and logit link function, using aggregated event and at-risk counts derived from pre- and post-diagnostic percentages (event = post − pre; at-risk = 100 − pre).

### 2.5. Ethical Consideration/Approval

This study was approved by the Turkish Ministry of Health on 27 March 2019 (approval number: 95741342-020-325).

## 3. Results

### 3.1. General Characteristics of the Lung Cancer Study Population (ICD-10 C34 Group) and MPM Study Group (ICD-10 FE MC45 Group)

A total of 74,835 patients diagnosed with LC were included in the study, of whom 21,174 (28.3%) were female. The mean age was 61.9 ± 15.1 years for females and 63.2 ± 12.6 years for males.

In addition, 1678 patients with MPM were included, comprising 764 females (45.5%) and 914 males (54.5%). The mean age was 61.9 ± 15.1 years for females and 62.4 ± 13.6 years for males.

### 3.2. Comorbidity Rates of the Study Population

In the LC group, the prevalence of comorbidities was compared between the pre- and post-diagnostic periods. The burden of comorbid conditions increased significantly after diagnosis in both females and males (*p* < 0.05). In the post-diagnostic period, the most common comorbidities observed in patients with LC were pneumonia, HT, and chronic COPD, with prevalence rates of 67.7%, 63.7%, and 55.9%, respectively. Compared with the pre-diagnostic period, the comorbidity risk in LC was highest for pulmonary embolism (OR = 2.88), ARF (OR = 2.65), and pneumonia (OR = 2.57) in the post-diagnostic period, respectively. (Table 1).

When comparing changes in comorbidity burden between sexes in the lung cancer group, male patients had higher frequencies of pulmonary embolism, pneumonia, pneumothorax, and coronary artery disease than females (respectively: OR = 3.06 vs. 2.54; OR = 2.76 vs. 1.89; OR = 2.82 vs. 2.33; OR = 1.65 vs. 1.49). In contrast, in the female lung cancer group, the prevalence of chronic renal failure was higher than in males (OR = 2.14 vs. 2.00), whereas acute renal failure was more prominent in the male patient group (OR = 2.64 vs. 1.94).

As shown in Table 2, in the MPM group, the prevalence of all evaluated comorbidities was significantly higher after diagnosis in both males and females (*p* < 0.05). In the post-diagnostic period, the most prevalent comorbidities in patients with MPM were hypertension (HT), pneumonia, and COPD, with rates of 66.9%, 65.2%, and 46.0%, respectively. Pneumothorax was infrequently observed (3.6%) in this group (Table 2).

In the MPM group, the greatest increases in comorbidity risk between the pre- and post-diagnostic periods were observed for acute renal failure (ARF) (OR = 5.95), phlebitis/venous thrombosis/thrombophlebitis (OR = 2.45), and chronic renal failure (CRF) (OR = 2.29). The smallest increase was observed for pleural effusion (OR = 1.45) (Table 2). In the gender-based comparison of comorbid conditions among patients with malignant pleural mesothelioma (MPM), the risk of both chronic renal failure (CRF) and acute renal failure (ARF) was higher in females compared to males (respectively: OR = 2.63 vs. 2.16 and OR = 6.80 vs. 5.44). Additionally, increased rates of COPD were observed in male patients within this group (OR = 1.93 vs. 1.81).

**Figure 1:**
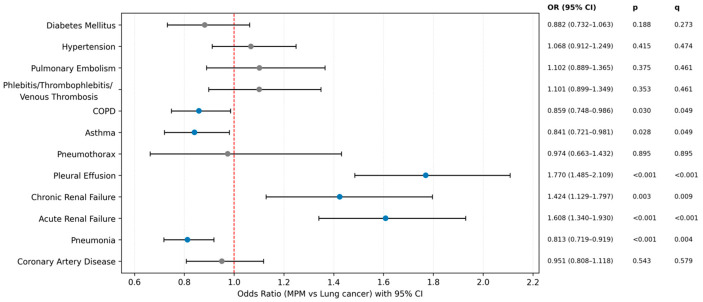
Sex- adjusted odds ratios (95% CI) for comorbidities in MPM and LC.

Figure 1 presents sex-adjusted odds ratios comparing comorbidities between MPM and LC. After false discovery rate (FDR) correction, pleural effusion (OR = 1.770, 95% CI 1.485–2.109; q < 0.001), acute renal failure (OR = 1.608, 95% CI 1.340–1.930; q < 0.001), and chronic renal failure (OR = 1.424, 95% CI 1.129–1.797; q = 0.009) were significantly more frequent in MPM. In contrast, COPD (OR = 0.859, 95% CI 0.748–0.986; q = 0.049), asthma (OR = 0.841, 95% CI 0.721–0.981; q = 0.049), and pneumonia (OR = 0.813, 95% CI 0.719–0.919; q = 0.004) were significantly less frequent in MPM. No statistically significant between-group differences were observed for diabetes mellitus, hypertension, pulmonary embolism, venous thrombosis-related diagnoses, pneumothorax, or coronary artery disease after FDR correction.

### 3.3. Comparative Results: LC Versus MPM

As shown in Table 3, there were no significant differences in the prevalence of diabetes mellitus (DM) and coronary artery disease (CAD) between the two cancer types in either the pre- or post-diagnostic periods (*p* > 0.05). However, when the two cancer groups were compared across diagnostic periods, the prevalence of hypertension (HT), pulmonary embolism (PE), deep venous thrombosis (DVT), pneumothorax, and pleural effusion was significantly higher in the MPM group (*p* < 0.05).

In contrast, asthma and chronic obstructive pulmonary disease (COPD) were more prevalent in the LC group than in the MPM group in both diagnostic periods. During the pre-diagnostic period, pneumonia and renal failure (ARF and CRF) were observed at comparable rates in both cancer types (*p* > 0.05). In the post-diagnostic period, pneumonia emerged as the most common comorbidity in patients with LC (*p* = 0.031), whereas renal failure was the most frequent comorbidity in the MPM group (*p* < 0.001 and *p* = 0.024, respectively).

## 4. Discussion

To the best of our knowledge, this is the first nationwide study from Turkey to comprehensively evaluate the burden of both pulmonary and extrapulmonary comorbidities in patients with LC and MPM. A key challenge in the management of comorbidity is the presence of pulmonary conditions prior to the diagnosis of lung cancer, which may contribute to the course of the disease. Furthermore, following diagnosis, the presence of comorbidities may adversely affect the patient’s quality of life and complicate clinical management, including delays or limitations in surgical and oncological treatment. Certain comorbid conditions may also negatively influence the disease course, potentially contributing to cancer progression and metastasis.

Our findings demonstrate that patients with both cancer types exhibit a substantial burden of comorbidities in the pre-diagnostic period, with a marked increase following diagnosis. A significant post-diagnostic rise in comorbid disease burden was observed, including COPD, asthma, pneumonia, pneumothorax, pulmonary embolism, deep venous thrombosis, hypertension, diabetes mellitus, renal failure, and coronary artery disease. While the prevalence of diabetes mellitus and coronary artery disease remained relatively stable across diagnostic periods, distinct patterns emerged between the two cancer types.

The observed differences in comorbidity patterns between LC and MPM likely reflect fundamental distinctions in tissue origin, etiologic exposures, and underlying pathophysiological mechanisms. LC originates from the bronchial or alveolar epithelium and directly involves the airways, lung parenchyma, and pulmonary vasculature. This results in a higher prevalence of airway-, vascular-, and infection-related comorbidities such as COPD, asthma, pneumonia, thromboembolic events, and cardiovascular disease, many of which are already present before diagnosis due to shared risk factors, particularly smoking. In contrast, MPM arises from pleural mesothelial cells and initially spares the lung parenchyma, leading predominantly to restrictive respiratory impairment driven by pleural inflammation, fibrosis, and effusions associated with asbestos exposure. While LC tends to exhibit a higher thromboembolic burden likely related to vascular involvement, hypoxia, and a prothrombotic tumor phenotype, MPM is characterized by more localized pleural involvement. Although the comorbidity burden increases after diagnosis in both diseases due to cancer and treatment-related systemic effects, the overall comorbidity profile remains strongly influenced by tumor biology and anatomical origin.

In the MPM group, comorbidities such as hypertension, pulmonary embolism, deep venous thrombosis, pneumothorax, pleural effusion, and renal failure (both acute and chronic) were more frequently observed, particularly in the post-diagnostic period. In contrast, in the LC group, COPD, asthma, and pneumonia were more prevalent. Notably, pneumonia and renal failure were observed at similar rates between the two cancer types in the pre-diagnostic period; however, after diagnosis, pneumonia became the most common comorbidity in LC, whereas renal failure predominated in MPM.

Cancer survival is influenced by multiple factors, including patient characteristics, histopathology, tumor stage at diagnosis, tumor–host interactions, and comorbidities. Comorbid conditions play a critical role in determining initial treatment strategies and the effectiveness of subsequent therapies. Previous studies have demonstrated that patients with breast cancer, prostate cancer, lymphoma, or lung cancer who have pre-existing comorbidities are more likely to receive less aggressive treatment modalities [12].

As previously reported, several comorbidities, including hypertension (HT), coronary artery disease (CAD), cerebrovascular disease, chronic obstructive pulmonary disease (COPD), and diabetes mellitus (DM), have a significant impact on cancer survival, including in the overall LC population [13,14].

Smoking, aging, and environmental exposures are key shared risk factors for lung cancer. COPD, in particular, constitutes a major global health burden and frequently coexists with lung cancer due to its shared etiopathogenesis, particularly smoking. It has been associated with an increased incidence of LC and may adversely affect treatment options and outcomes, ultimately leading to reduced overall survival [15]. Notably, COPD is among the most common comorbidities in patients with lung cancer.

Consistent with the literature, our findings demonstrated that COPD, asthma, and pneumonia were among the most frequently observed comorbid conditions. Oxidative stress and chronic inflammation are considered key mechanisms linking COPD and lung carcinogenesis. Furthermore, COPD may limit the feasibility of radical treatment options, thereby negatively influencing clinical outcomes [16].

Cardiovascular comorbidities, including HT, CAD, peripheral vascular disease, arrhythmias, and abdominal aortic aneurysm, were also highly prevalent in patients with cancer. In the present study, particular attention was given to CAD, given its high prevalence in lung cancer populations, which has been reported to range from 12.9% to 43% in previous studies [17,18].

In line with these findings, the prevalence of CAD in our cohort increased from 27.4% in the pre-diagnostic period to 37.6% following diagnosis.

Hypertension and lung cancer are among the most prevalent and burdensome chronic diseases worldwide, and their coexistence presents complex challenges in both clinical practice and biomedical research. Emerging evidence suggests shared pathogenic mechanisms, including chronic inflammation, oxidative stress, vascular dysfunction, and metabolic dysregulation. Environmental factors such as air pollution, tobacco smoke, and heavy metal exposure may further contribute to genetic and epigenetic alterations, increasing susceptibility to both conditions [19,20].

In our study population, hypertension was one of the most common comorbidities observed in the post-diagnostic period in patients with LC. However, when compared across cancer types, the prevalence of HT was higher in the MPM group. A deeper understanding of the shared genetic and epigenetic mechanisms underlying hypertension and thoracic malignancies may provide novel opportunities for improved risk stratification, early detection, and the development of targeted therapeutic strategies. Future research should focus on integrating genetic screening with environmental risk assessment to advance precision medicine approaches for individuals at increased risk.

In patients with cancer, the poorer prognosis associated with coexisting diabetes mellitus (DM) may be attributed to biological mechanisms such as hyperglycemia, hyperinsulinemia, and chronic inflammation, all of which promote tumor proliferation and metastasis. Additional factors include the association with cancer treatment and reduced responsiveness to cancer therapy in patients with DM. Therefore, strict glycemic control should be emphasized during cancer treatment and throughout the disease course [21,22].

In our study, the prevalence of DM increased from 19.9% in the pre-diagnostic period to 27.7% after diagnosis. By contrast, Islam et al. reported a slightly lower prevalence of 15.7% among patients with LC during the disease course [18].

A nationwide study in Taiwan found that patients with LC and pulmonary comorbidities had lower overall survival than those without pulmonary disease. Previous studies have also reported the negative impact of asthma and COPD on survival across different lung cancer subtypes [23].

Patients with cancer are at increased risk of thrombotic events, either as a paraneoplastic phenomenon or due to hypercoagulable states associated with reduced levels of protein S, protein C, and antithrombin III, as well as elevated cytokine levels [24]. In a cohort of 100 patients, De León et al. reported a venous thromboembolism (VTE) incidence of 32% among patients undergoing surveillance after pleurectomy for mesothelioma. Notably, up to 33% of patients with deep venous thrombosis (DVT) were asymptomatic at the time of diagnosis, leading the authors to recommend routine screening for early detection and prevention of progression to symptomatic or fatal pulmonary embolism (PE) [25].

Thromboembolic events (TEEs), including DVT, PE, arterial thrombosis, and myocardial infarction, frequently occur during the disease course and have been reported to reach rates of up to 27.7% in MPM [26]. In addition, cancer treatments including chemotherapy, hormonal therapy, antiangiogenic agents, radiotherapy, and surgical interventions may further increase the risk of thrombogenesis [26].

In a previous study by Köksal et al., the incidence of TEEs in patients with MPM was reported as 7.9%. Consistent with these findings, in our MPM cohort, the prevalence of PE ranged from 4.8% to 10.3% and DVT from 4.7% to 10.8% across the pre- and post-diagnostic periods [27]. Furthermore, our results showed that PE and DVT were more prevalent in the MPM group than in the LC group, suggesting a higher thrombosis risk in patients with MPM.

In our study population, pneumonia was the most common comorbidity in patients with LC during the post-diagnostic period, whereas renal failure predominated in the MPM group. Supporting these findings, a national cohort study from France by Chouaid et al. reported that comorbidities in patients with MPM were associated with poor prognosis. Specifically, advanced age (>70 years), chronic renal failure, chronic respiratory failure, and the absence of pemetrexed treatment were identified as significant predictors of worse outcomes [28].

Our findings demonstrate that patients in both cancer groups already exhibited a substantial burden of comorbidities prior to diagnosis, which increased further in the post-diagnostic period. Similarly, a nationwide study from Sweden by Linden et al. reported that patients with LC had poorer health status even before diagnosis, which contributed to worse clinical outcomes. Conditions that were prevalent prior to diagnosis, such as hematologic disturbances (e.g., anemia, neutropenia) and respiratory disorders (e.g., pneumonia and respiratory infections), were also observed with higher frequency following diagnosis, reflecting their persistence and progression over time [29].

Borg et al. further suggested that a shared genetic predisposition may underlie both lung cancer and lifestyle-related comorbidities. In addition, lung cancer and its treatment may exacerbate or unmask previously undiagnosed chronic pulmonary conditions [30].

When comparing changes in comorbidity burden between sexes in the lung cancer group, male patients had higher frequencies of pulmonary embolism, pneumonia, pneumothorax, and coronary artery disease than females. These findings suggest that men may have a greater susceptibility to thromboembolic and cardiopulmonary complications. This difference may be partly explained by higher rates of smoking exposure, as well as a greater prevalence of underlying COPD and cardiovascular diseases in male patients. Additionally, sex-related differences in tumor biology, inflammatory response, and treatment-related complications may also contribute to this disparity. Therefore, closer monitoring and early preventive strategies for these specific comorbidities may be particularly important in the clinical management of male patients.

In contrast, in the female lung cancer group, the prevalence of chronic renal failure was higher than in males, whereas acute renal failure was more prominent in the male patient group. Overall, renal impairments appear as significant comorbid conditions in lung cancer patients of both sexes, with chronic renal failure being particularly more evident among women. In previous literatüre it was reported that genetic and biological differences between men and women could explain the disparity in incidence and mortality of lung cancers; much remains unanswered. The degree which gender-associated habits, genetics and environmental exposure to carcinogenic agents participate in these differences is unclear [31].

Previous study of Barsky et al. underlined that despite the observed survival advantage in female patients with malignant pleural mesothelioma, the lower rates of surgical and chemotherapeutic interventions suggest the presence of gender-based disparities in access to care, highlighting the need for further investigation into the underlying biological and healthcare system-related factors [32]. Our findings suggest that sex-specific differences in comorbidity patterns among MPM patients may reflect distinct underlying biological susceptibilities and exposure histories. The higher burden of renal complications in females could be related to differences in baseline renal vulnerability, treatment tolerance, or healthcare utilization patterns, whereas the increased prevalence of COPD in males likely reflects greater cumulative exposure to respiratory risk factors such as smoking or occupational hazards. Accordingly, these disparities highlight the importance of adopting a sex-sensitive approach in the clinical assessment and management of comorbidities in MPM patients.

## 5. Conclusions

In conclusion, our findings indicate that both LC and MPM are associated with a significant burden of comorbidities already present in the pre-diagnostic period, with a further increase following diagnosis. Consequently, patients with these malignancies face the dual challenge of managing both their primary disease and multiple coexisting conditions throughout the disease course.

Comprehensive assessment of comorbidities is essential for guiding clinical decision-making, anticipating disease progression, and optimizing treatment strategies. Moreover, awareness of comorbidity patterns may help clinicians and caregivers plan more effective follow-up and management strategies for these high-risk patient populations. Early identification, monitoring, and targeted management of comorbid conditions may improve patient outcomes and support more efficient use of healthcare resources.

Future studies incorporating appropriate control groups, longitudinal follow-up, and adjustment for confounding factors are needed to clarify the clinical significance of these associations and to determine whether specific comorbidity patterns may have implications for risk stratification, surveillance, or management strategies.

### Strengths, Implications, and Limitations of the Study

The major strengths of this study include its large, population-based sample, the comparative analysis of LC and MPM, and its focus on healthcare service utilization. To the best of our knowledge, this is the first study based on nationwide data from Turkey to comprehensively evaluate the burden of pulmonary and extrapulmonary comorbidities in patients with LC and MPM, with a specific focus on both pre- and post-diagnostic periods. Furthermore, the study covers the period from 2015 to 2018, representing the pre–COVID-19 era, thereby avoiding potential confounding effects related to the pandemic.

A key clinical challenge highlighted by our findings is the presence of pulmonary comorbidities prior to the diagnosis of lung malignancies, which may contribute to the course of the diagnostic period. In the post-diagnostic period, comorbidities may further complicate disease management by impairing quality of life, increasing healthcare costs, and limiting access to surgical and oncological treatments. In addition, certain comorbid conditions may adversely influence the course of cancer, potentially contributing to disease progression and metastasis. Awareness of comorbidity burden may therefore assist clinicians and caregivers in anticipating adverse outcomes and optimizing follow-up and management strategies.

Despite growing evidence on the impact of comorbidities in thoracic malignancies, several important gaps remain. Most previous studies have focused on comorbidity burden at a single time point, without distinguishing between pre- and post-diagnostic periods. In addition, comparative data between LC and MPM are limited, particularly within the same population. Furthermore, large-scale, nationwide analyses using real-world electronic medical records are scarce. As a result, the temporal evolution and comparative burden of comorbidities in LC and MPM remain insufficiently characterized.

However, several limitations should be acknowledged. First, the administrative dataset lacked detailed clinical information, including histopathological findings, tumor stage, treatment protocols, performance status, and survival outcomes. Second, important potential confounders such as smoking history, socioeconomic status, and treatment-related variables were not available. Consequently, the primary analyses are based on crude (unadjusted) comparisons, and the regression models were adjusted only for sex. Moreover, because person-time data are unavailable, post-diagnostic findings should be interpreted as cumulative rates over the observation period rather than incidence rates. Finally, the dataset did not allow for verification of potential inaccuracies or incomplete records, which may have introduced information bias. Another methodological limitation of this study is that odds ratios were calculated using aggregated percentage data rather than individual-level paired observations. Additionally, an important limitation of this study was the asymmetry between the pre-diagnostic and post-diagnostic observation periods. The longer follow-up duration in the post-diagnostic phase might have increased the likelihood of detecting comorbidities, independent of true biological differences. This temporal imbalance could therefore lead to an apparent inflation in post-diagnostic comorbidity prevalence. However, the duration of the post-diagnostic period was determined by the available follow-up data within the national database. This limitation should be considered when interpreting pre–post comparisons. Another important limitation of this study is the potential misclassification of post-diagnostic comorbidities. Certain conditions identified after diagnosis, such as pneumonia, acute renal failure, pulmonary embolism, and pleural effusion, may represent complications of the underlying malignancy or its treatment rather than independent comorbidities. Due to the use of ICD-10 codes within a national administrative database, it was not possible to reliably distinguish between pre-existing comorbidities, cancer-related complications, and treatment-associated toxicities. This may have resulted in differential misclassification, particularly in the post-diagnostic period, potentially leading to an overestimation of post-diagnostic associations. As mentioned, patients diagnosed with cancer are candidates for more intensive medical follow-up, including imaging studies, laboratory testing, and more frequent clinical evaluations. This increased level of healthcare visits may lead to the identification of previously undiagnosed or subclinical conditions, thereby creating an ascertainment asymmetry between pre- and post-diagnosis periods. Consequently, part of the observed increase in comorbidity prevalence following cancer diagnosis may reflect enhanced detection rather than true incident disease. While using a large national database provides substantial statistical power, we recognize that the lack of granular clinical data and standardized screening intensity limits our ability to fully account for this bias. This situation constitutes a major limitation of our study We acknowledge that calculating odds ratios from aggregated percentages rather than individual-level paired data is also a limitation of our analytical approach. In our study, several post-diagnostic comorbid conditions indeed have relatively high prevalences (ranging from 20% to 68%), which may lead to such overestimation. However, due to the design of our study and the use of aggregated data, we were unable to directly calculate the prevalence ratios. Therefore, ORs were used as a measure of association, consistent with similar large-scale observational studies. Future studies incorporating matched control groups or longitudinal designs with standardized surveillance protocols may help conduct better research.

## Figures and Tables

**Table 1:** Comparison of lung cancer group in terms of comorbid conditions pre and post diagnostic periods.

	Total (n = 74,835)			Male (n = 53,661)			Female (n = 21,174)		
Variables	Pre (%)	Post (%)	OR (95% CI)	Pre (%)	Post (%)	OR (95% CI)	Pre (%)	Post (%)	OR (95% CI)
Pulmonary embolism	2.9	7.8	2.88 (2.74–3.03)	2.7	7.6	3.06 (2.87–3.25)	3.5	8.5	2.54 (2.33–2.78)
Asthma	28.2	41.0	1.73 (1.69–1.81)	27.1	37.3	1.83 (1.78–1.89)	31.1	50.1	1.70 (1.63–1.76)
COPD	39.0	55.9	1.94 (1.89–2.00)	44.4	63.1	2.05 (2.00–2.10)	25.0	39.9	1.99 (1.91–2.07)
Pneumothorax	1.0	2.8	2.56 (2.16–3.05)	1.0	2.9	2.82 (2.57–3.09)	1.0	2.3	2.33 (1.98–2.74)
Pleural effusion	3.5	8.6	2.45 (2.24–2.69)	3.2	8.0	2.80 (2.65–2.97)	4.4	10.2	2.76 (2.58–2.94)
Pneumonia	44.8	67.7	2.57 (2.53–2.64)	46.5	70.6	2.76 (2.70–2.82)	40.3	60.4	1.89 (1.70–2.11)
Diabetes mellitus	17.0	21.7	1.55 (1.52–1.59)	17.0	21.5	1.55 (1.50–1.59)	17.1	22.2	1.56 (1.50–1.63)
Hypertension	53.4	63.7	1.54 (1.52–1.56)	50.6	60.5	1.56 (1.52–1.59)	60.5	71.4	1.50 (1.43–1.56)
Chronic renal failure	3.7	7.0	1.98 (1.89–2.11)	3.8	7.1	2.00 (1.92–2.11)	3.4	6.9	2.14 (1.98–2.32)
Acute renal failure	1.6	4.8	2.65 (2.44–2.88)	1.5	4.7	2.64 (2.31–5.00)	1.9	5.0	1.94 (1.77–2.13)
Coronary Artery diseases	27.4	37.6	1.66 (1.63–1.69)	26.5	36.5	1.65 (1.60–1.69)	29.8	40.6	1.49 (1.43–1.56)

OR: Odds Ratio, CI: Confidence interval. COPD: chronic obstructive pulmonary disease.

**Table 2:** Comparison of pre- and post-diagnostic comorbid conditions in MPM patients.

	Total (n = 1678)			Male (n = 914)			Female (n = 764)		
Variables	Pre (%)	Post (%)	OR (95% CI)	Pre (%)	Post (%)	OR (95% CI)	Pre (%)	Post (%)	OR (95% CI)
Pulmonary embolism	4.8	10.3	2.25 (1.71–2.96)	4.7	10.7	2.43 (1.68–3.53)	5.0	9.7	2.25 (1.71–2.96)
Deep venous thrombosis	4.7	10.8	2.45 (1.86–3.22)	4.8	10.6	2.35 (1.62–3.40)	4.6	11.0	2.57 (1.71–3.87)
Asthma	25.4	36.4	1.68 (1.45–1.98)	20.8	32.1	1.80 (1.46–2.22)	30.9	41.6	1.60 (1.29–1.97)
COPD	31.6	46.0	1.85 (1.60–2.13)	38.3	54.5	1.93 (1.60–2.32)	23.6	35.9	1.81 (1.45–2.27)
Pleural effusion	30.9	39.4	1.45 (1.23–1.67)	33.3	42.1	1.46 (1.21–1.77)	28.1	36.1	1.44 (1.16–1.79)
Pneumothorax	2.0	3.6	1.82 (1.19–2.79)	2.4	4.0	1.71 (1.01–2.92)	1.6	3.1	2.03 (1.01–4.09)
Pneumonia	46.1	65.2	2.19 (1.90–2.51)	49.8	68.4	2.18 (1.80–2.64)	41.8	61.4	2.22 (1.81–2.72)
Diabetes mellitus	21.2	28.5	1.48 (1.26–1.73)	18.2	25.1	1.51 (1.20–1.89)	24.9	32.6	1.46 (1.17–1.83)
Hypertension	56.2	66.9	1.57 (1.37–1.81)	51.3	63.6	1.66 (1.34–2.00)	62.0	70.8	1.48 (1.20–1.84)
Chronic Renal Failure	3.9	8.5	2.29 (1.70–3.10)	5.0	10.3	2.16 (1.50–3.12)	2.5	6.3	2.63 (1.53–4.52)
Acute Renal Failure	1.7	9.5	5.95 (3.98–8.90)	2.0	9.8	5.44 (3.25–9.10)	1.4	9.0	6.80 (3.57–12.95)
Coronary Artery Disease	26.5	36.4	1.59 (1.37–1.84)	29.4	39.3	1.55 (1.28–1.88)	22.9	32.9	1.65 (1.31–2.07)

OR: Odds Ratio, CI: Confidence interval. COPD: chronic obstructive pulmonary disease.

**Table 3:** Comparison of comorbidities in lung cancer and mesothelioma groups according to diagnostic periods.

Variable	Pre LC (%)	Pre MPM (%)	χ^2^; *p*	OR (95% CI)	Post LC (%)	Post MPM (%)	χ^2^; *p*
Pulmonary embolism	2.9	4.8	χ^2^ = 22.502; *p* < 0.001	1.72 (1.15–1.58)	7.8	10.3	χ^2^ = 13.433; *p* < 0.001
Deep venous thrombosis	3.3	4.7	χ^2^ = 9.508; *p* = 0.002	1.43 (1.14–1.80)	8.9	10.8	χ^2^ = 7.123; *p* = 0.008
COPD	39.0	31.6	χ^2^ = 37.845; *p* < 0.001	1.38 (1.25–1.54)	55.0	46.0	χ^2^ = 64.627; *p* < 0.001
Asthma	28.2	25.4	χ^2^ = 6.300; *p* = 0.012	1.15 (1.03–1.29)	41.0	36.4	χ^2^ = 14.238; *p* < 0.001
Pneumothorax	1.0	2.0	χ^2^ = 15.884; *p* < 0.001	2.00 (1.41–2.83)	2.8	3.6	χ^2^ = 3.961; *p* = 0.047
Pleural effusion	3.5	30.9	χ^2^ = 3097.156; *p* < 0.001	12.18 (10.91–13.61)	8.6	39.4	χ^2^ = 1840.491; *p* < 0.001
Pneumonia	44.8	46.1	χ^2^ = 1.151; *p* = 0.283	NS	67.7	65.2	χ^2^ = 4.642; *p* = 0.031
Diabetes mellitus	19.9	21.2	χ^2^ = 1.830; *p* = 0.176	NS	27.7	28.5	χ^2^ = 0.473; *p* = 0.492
Hypertension	53.4	56.2	χ^2^ = 5.207; *p* = 0.022	1.15 (1.04–1.27)	63.7	66.9	χ^2^ = 6.917; *p* = 0.009
Chronic Renal Failure	3.7	3.9	χ^2^ = 0.171; *p* = 0.679	NS	7.0	8.5	χ^2^ = 5.068; *p* = 0.024
Acute Renal Failure	1.6	1.7	χ^2^ = 0.141; *p* = 0.708	NS	6.8	9.5	χ^2^ = 18.430; *p* < 0.001
Coronary Artery Disease	27.4	26.5	χ^2^ = 0.742; *p* = 0.389	NS	37.6	36.4	χ^2^ = 1.075; *p* = 0.300

χ^2^: Chi-square, OR: Odds Ratio, CI: Confidence interval, NS: Not Significant. COPD: chronic obstructive pulmonary disease.

## Data Availability

The data are held by the Turkish Ministry of Health. Access is subject to institutional permissions and legal and ethical restrictions.
